# Hippocampal Dosimetry and Mnemonic Function Changes After Stereotactic Irradiation of Cavernous Sinus Meningiomas

**DOI:** 10.7759/cureus.20252

**Published:** 2021-12-07

**Authors:** Mikhail V Galkin, Gleb V Danilov, Maria Y Kaverina, Yulia V Strunina, Olga A Krotkova

**Affiliations:** 1 Radiotherapy Department/Radiation Oncology, N.N. Burdenko National Medical Research Center for Neurosurgery, Moscow, RUS; 2 Laboratory of Biomedical Informatics and Artificial Intelligence, N.N. Burdenko National Medical Research Center for Neurosurgery, Moscow, RUS; 3 Rehabilitation Department/Neuropsychology, N.N. Burdenko National Medical Research Center for Neurosurgery, Moscow, RUS

**Keywords:** hippocampal dosimetry, pattern separation, hippocampus, memory, radiotherapy, cavernous sinus meningioma

## Abstract

Introduction: It is believed that hippocampal exposure plays a major role in the development of memory disorders after cranial irradiation. This effect is evident in whole-brain irradiation and is less certain in local irradiation of intracranial targets. The present study aims to clarify the dosimetric features and dynamics of memory functions after local irradiation of the hippocampus when treating cavernous sinus meningiomas.

Methods: The study included 28 patients (24 females and 4 males) with cavernous sinus meningiomas diagnosed according to typical clinical and radiological findings. The mean age was 52 years (30-65 years). Stereotactic radiotherapy in standard fractionation regimen (54 Gy total dose) was the primary treatment in all patients. Patients underwent memory testing (ability to reproduce and recognize) using a previously developed and validated methodology at standard time points: before the start of radiotherapy, at the end of the course, and 6 and 12 months after treatment. Hippocampal dose, dynamics of memory function, and their possible relationship were evaluated.

Results: In total, 28 cavernous sinus meningiomas (15 left-sided and 13 right-sided) were treated. The mean target volume was 24.0 ccm (8.2 ccm to 53.8 ccm). Twelve months after radiotherapy, there was an increase in the median total number of recognition errors from 6.5 [4;11] to 9.5 [5;12], p=0.025, the median number of "old-similar" errors from 2 [1;3.25] to 3 [1.75;5], p=0.021, and the median number of "similar-old" errors from 3 [1;5] to 5.5 [3;7], p<0.001. The number of reproduction errors did not increase. A moderate correlation (p = 0.03, correlation coefficient = 0.41) was found between the dose to 10% of the ipsilateral hippocampus and the total number of reproduction errors at the end of the course. No other significant correlations were found at the end of radiotherapy and six and 12 months after it.

Conclusion: Thus, even partial lateralized exposure of the hippocampus during irradiation of the cavernous sinus meningiomas affects its function in the form of specific pattern separation type disturbances, which are detected as early as 12 months after the impact. The hippocampus in this treatment should be considered as a critical structure whose sensitivity to irradiation requires additional assessment.

## Introduction

Radiotherapy is one of the principal treatments for primary and secondary intracranial neoplasms. At the same time, there is a certain risk of causing brain toxicity. In particular, deterioration of verbal and spatial memory, attention, counting, and the ability to solve new tasks are possible [1].

A key mechanism for the development of cognitive impairment in patients after cranial radiotherapy is currently thought to be a negative impact on the hippocampus [2,3]. The crucial role of the hippocampus in providing mnemonic function has been confirmed in many studies. It was demonstrated in experiments that morphological changes, including apoptosis, are detected in the hippocampus of young rats already in a few hours after exposure to a single dose of 5-30 Gy [4,5]. Behavioral tests in rodents after hippocampal irradiation also revealed cognitive abnormalities [6]. Children who had received radiation treatment as part of research protocols and underwent cognitive assessments years after exposure were found to have lower IQ at doses greater than 45 Gy per temporal lobe [7,8]. A study of cognitive function in patients with benign cerebral gliomas revealed that when the equivalent dose of 7.3 Gy on 40% of the hippocampal volume is exceeded, long-term cognitive impairment is observed [9]. When the dose to the hippocampus is intentionally restricted during whole-brain irradiation for metastatic lesions, the rate of memory impairment decreases from 30% to 7% [10].

Nevertheless, the treatment of patients with CNS pathology much more often utilizes local irradiation of the tumor rather than whole-brain irradiation (WBI). Among others in neuro-oncology, there is a rather large group of patients with neoplasms of the sellar region-meningiomas, adenomas, chordomas, craniopharyngiomas, etc. The incidence of pituitary and stalk neoplasms alone is 17.9% among all primary CNS tumors, without taking into account meningeal tumors of the region [11]. Patients with neoplasms in this area are frequent candidates for radiotherapy because the deep location and complex anatomy in many cases prevent the complete resection. At the same time, hippocampi receive a significant dose during radiotherapy of these tumors because of their proximity. The risks of memory impairment in partial and lateralized radiation exposure to the hippocampus have been much less investigated than in WBI. In addition, most of the studies on partial hippocampal irradiation fail to eliminate the effect of other factors such as surgical treatment (in gliomas, craniopharyngiomas), hormonal disorders (craniopharyngiomas, pituitary adenomas), the infiltrative effect of the tumor itself on the brain (in gliomas) [7-9].

The present study examines the dynamics of the mnemonic function in patients with meningiomas of the cavernous sinus, who received only radiotherapy. It is assumed that the benign nature of meningiomas, non-infiltrative, slow growth minimizes the influence of other factors on memory function. 

## Materials and methods

The study was approved by an ethics committee of the N.N. Burdenko National Medical Research Center for Neurosurgery (approval number - 05/2017). The study included 28 patients with cavernous sinus meningiomas. There were 24 women and four men. The average age was 52 years (range, 30-65 years). In all cases, this course of radiotherapy was the primary treatment for the tumor. The diagnosis of "benign meningioma" was established in all cases on the basis of typical clinical and neuroimaging findings. All patients underwent topometric head MRI (axial T2 images [slice thickness 2 mm], axial three-dimensional spoiled gradient recalled acquisition in steady state [3D SPGR] enhanced and non-enhanced [slice thickness 1 mm]) before the treatment. Target and hippocampal contouring was performed in the iPlan planning system (BrainLab, Munich, Germany) for accurate volume estimation and dosimetry. Hippocampal contouring was performed according to the Radiation Therapy Oncology Group (RTOG) 0933 protocol and the work of Chera et al. on axial images, sequentially manually on each slice using all available modalities [12].

All patients underwent stereotactic conformal radiotherapy using a photon beam according to the standard technique on Novalis LinAc ("Varian" [Varian, Crawley, UK] and "BrainLab" [BrainLab, Munich, Germany]) (6 MeV) equipped with micromultileaf collimator. Treatments were performed in the radiotherapy department of the N.N. Burdenko National Medical Research Center of Neurosurgery, named after the academician N.N. Burdenko. Irradiation was performed using six static conformal beams. Hippocampal contouring was performed after the irradiation plan had been created; therefore, no special dose limitation on the hippocampus was performed.

Irradiation was performed in the standard regimen with a fraction size of 1.8 Gy up to a total dose of 54 Gy. Doses to 10%, 30%, and 50% of the volume of each hippocampus and both hippocampi were assessed. Separately, the dose to 40% of both hippocampi was assessed with EQD2 (equivalent to 2 Gy fractions with α/β=2) recalculated according to Gondi et al. [9]. This dose is most often discussed as a formal restriction in partial hippocampal irradiation during the treatment of intracranial neoplasms.

Memory assessment was performed at fixed time points. The first point corresponded to the test performed before the beginning of radiotherapy. The second point was immediately after the end of the treatment (as a rule, 45 days after the first point). The third and the fourth points were located six and 12 months after the end of radiotherapy, respectively.

We used the original “eye tracking-attention-memory” (ETAM) methodology developed by O.A. Krotkova, which implied the recording of eye movements during visual attention and memory testing [13]. Five stimuli with the instruction "look at them carefully and remember" were sequentially presented to the subject on the screen. Each stimulus consisted of three color pictures arranged in a row (a triplet of pictures). The exposure time was 10 seconds for one triplet.

The free recall of the stored stimuli was performed 10 minutes after the presentation was finished. Reproduction errors were assessed. After another 15 minutes, the procedure of recognizing the stimulus material was performed. This time, separate images appeared on the screen in a pseudo-random order; some of these were identical to those shown at the first stage; some were slightly different in details, color, or location in the visual field; there were also completely new images (distractors) not related to the previously shown ones. At this stage, the stimulus material consisted of 30 images: 15 previously seen objects, 10 images similar to the lateral stimuli in the triplets shown at the first stage, and five new distractors previously not shown. In this test for recognition, the subject reported whether the image was seen previously, or it was similar to that seen previously, or it was new, not seen previously. The total number of recognition errors was estimated, as well as the frequency of individual error types. When describing patients' errors in the test of distinction of similar stimuli, the first is the type of stimulus ("Similar" - stimulus similar to a previously presented, "Old" - previously presented to the patient, "Novel" - new stimulus), the second is the response of the subject according to how he assessed the stimulus ("Similar", "Old", "Novel"). At each point during the study, the subject was shown a new set of stimuli each time.

Statistical analysis was performed using the free statistical programming language and software environment R (www.r-project.org, version 3.5.0). Mann-Whitney nonparametric test was used to assess the statistical significance of differences in the distribution of numerical values in two independent groups, and Wilcoxon nonparametric test was used in paired observations. The Spearman correlation coefficient was calculated to analyze the correlation between the two numerical values. Differences in the distributions of categorical variables were assessed using Chi-square and Fisher's exact test. Differences or correlations were considered statistically significant at the p<0.05 level.

## Results

In total, 28 cavernous sinus meningiomas were treated in 28 patients. In 15 cases, the tumor had a left-sided location, and in 13 cases, a right-sided location. The average target volume was 24.0 ccm (range, 8.2 ccm to 53.8 ccm). The mean value of the Karnofsky index at the beginning of treatment was 84 (range, 80 - 90). The functional status of the patients was mainly determined by visual and oculomotor disorders. The value of the Karnofsky index did not change during the observation period.

The mean doses to 10%, 30%, and 50% of the ipsilateral hippocampal volume were 40.0 Gy (range, 11.0-53.8 Gy), 29.3 Gy (range, 9.2-44.3 Gy), and 20.1 Gy (range, 4.5-35.8 Gy), respectively. The mean doses per 10%, 30%, and 50% contralateral hippocampal volume were 13.8 Gy (range, 3.2-32.3 Gy), 9.7 Gy (range, 1.9-20.1 Gy), and 8.0 Gy (range, 1.4-16.2 Gy), respectively. The mean doses to 10%, 30%, and 50% of total hippocampi volume were 33.9 Gy (range, 9.9-48.6 Gy), 18.6 Gy (range, 7.7-31.7 Gy), and 13.9 Gy (range, 7.1-23.4 Gy), respectively. The mean EQD2 value (with α/β=2) for 40% of both hippocampal volumes was 8.7 Gy (4.0-16.3 Gy). In 17 (60.7%) cases, the EQD2 value exceeded 7.3 Gy.

The following results were obtained when analyzing the dynamics of the errors of reproduction and recognition at time points two, three, and four. There was no increase in the total number of errors of reproduction.

**Figure 1:**
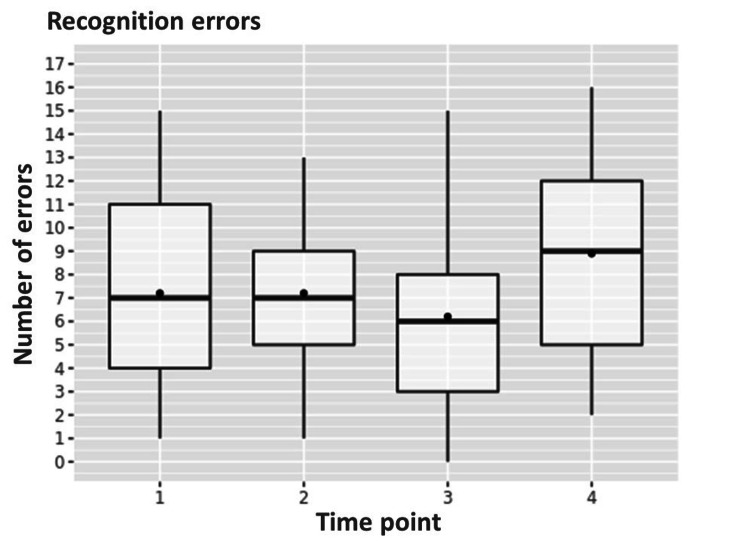
Dynamics of the total quantity of recognition errors from the first to the fourth time point

A significant increase in total recognition errors was observed from a median of 6.5 [4;11] points to a median of 9.5 [5;12] (p=0.025) from the first to the fourth time point (Figure 1).

**Figure 2:**
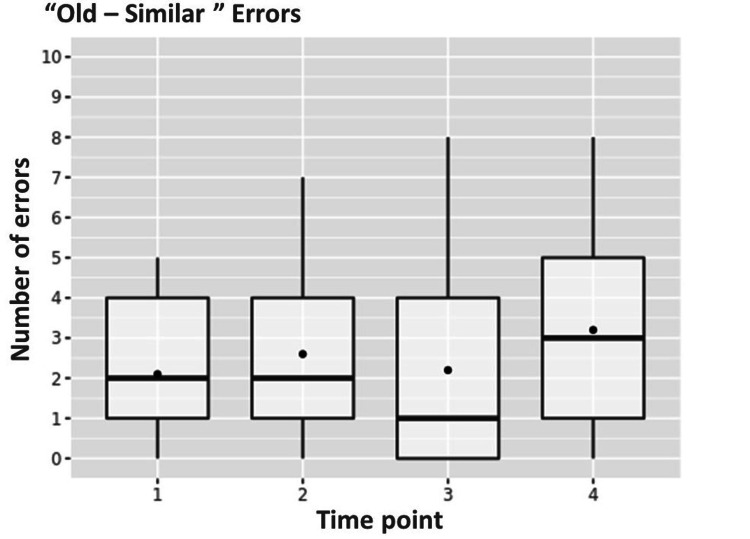
Dynamics of "old-similar" type of errors from the first to the fourth time point

At the same time interval, there was a significant increase in "old-similar" errors when the patient regarded a previously seen stimulus as similar to it, from a median of 2 [1;3.25] to a median of 3 [1.75;5] (p=0.021) (Figure 2).

**Figure 3:**
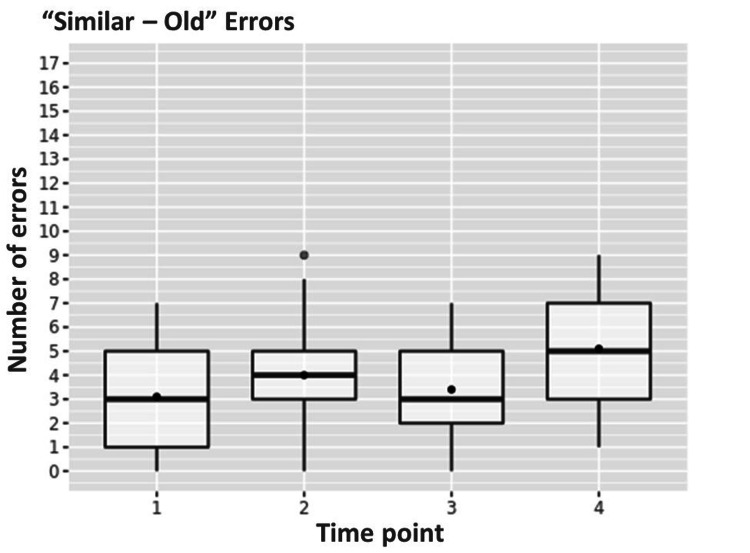
Dynamics of "similar-old" type of errors from the first to the fourth time point

There was also a significant increase from the first to the fourth point in the “similar-old” errors, from a median of 3 [1;5] to a median of 5.5 [3;7] (p<0.001) (Figure 3). 

No significant increase in other types of errors was detected. The correlation between the number of errors of different types and the dose to the hippocampus at different time points was studied. A correlation (p = 0.03, correlation coefficient = 0.41) was found between the dose to 10% of the ipsilateral hippocampus and the total number of reproduction errors for the second point (at the end of the course of radiation therapy).

No other significant correlations were found at point two for other types of errors and different hippocampal doses. Neither significant correlations of different error types and hippocampal doses at points three and four were detected. Among other things, we failed to detect a correlation between the number of errors at time points two, three, and four and EQD2 on 40% of both hippocampi.

## Discussion

This research is the first to clarify the significance of only partial irradiation of the human hippocampus in the standard fractionation mode, minimizing the influence of other factors, such as surgery, chemotherapy, tumor infiltration, post-therapy changes in the tumor [7-9]. It is also important that the study includes adult patients and is not associated with hypersensitivity of the developing brain to radiation exposure [7,8].

The study showed that during irradiation of relatively small (mean target volume - 24.0 cсm [8.2-53.8 cm^3^]) cavernous sinus meningiomas with standard fractionation, the dose in the hippocampus determines an increased risk of memory disturbances formation in most patients. Indeed, in 17 (60.7%) cases the EQD2 value (with α/β=2) exceeded 7.3 Gy in 40% of both hippocampi, which according to the study by Gondi et al. determines a higher risk [9]. Also, in most cases, the maximum dose to the hippocampus of 6 Gy or 16 Gy (which is used in some papers as one of the risk assessment criteria for WBI) was exceeded, as the average dose to 10% of the ipsilateral hippocampus in the current study was 40.0 Gy (range, 11.0-53.8 Gy) [14]. This determines, on the one hand, the potential risks that need to be taken into account when planning the radiation treatment of various neoplasms of the sellar area, such as meningiomas, adenomas, craniopharyngiomas, etc. On the other hand, it substantiates the need for research on this problem.

The dynamic assessment of memory functions revealed a significant change one year after the radiation treatment. At the same time, only specific components of memory suffered. No changes in the free reproduction of stimuli were revealed. However, there was a significant increase in recognition errors, namely errors of distinction between "seen" and "similar" stimuli, which is interpreted in the literature as a pattern separation error. It is believed that these types of errors may be associated with a suppression of neurogenesis in the hippocampus [15,16]. The detection of these specific changes in memory is associated with the chosen method of testing by the method of ETAM [13]. The memory changes detected were subclinical. Patients noted no changes in their daily activities. There were no changes in functional activity in the Karnofsky index.

In our study, the detectable changes in memory function were determined only 12 months after the completion of radiotherapy. This correlates with previously published data that the effects of radiotherapy can manifest over time and the severity of disturbances can increase along the way [17]. In many studies, cognitive impairment was determined years after radiotherapy [7,8]. It is likely that a longer follow-up of the selected group of patients will reveal more significant changes.

A correlation analysis between the hippocampal doses and the frequency of different types of errors at different time points did not provide the expected results. The correlation between the severity of cognitive impairment and dose has been found in several works. In contrast, we were only able to identify a moderate relationship between the number of errors in reproduction at the 2nd point and the dose to the 10% ipsilateral hippocampus (p = 0.03, correlation coefficient = 0.41). No other correlations, including with the determination of load by analogy with Gondi's work, could be detected [9]. One possible reason for this is an insufficiently long follow-up period, as according to the literature the severity and frequency of the impairment increase over time [17]. In most of the studies, the follow-up period was one to several years after irradiation [7-10,17]. In addition, other negative factors (surgery, chemotherapy, tumor infiltration, hormonal disturbances) may increase the effect of ionizing radiation and consequently produce more pronounced cognitive effects, which will increase the possibility of detecting correlations.

Thus, up to 12 months after fractionated irradiation of meningiomas of the cavernous sinus, mild negative mnestic effects of limited practical value are noted. Further evaluation is required to determine the long-term effects of such treatment.

## Conclusions

Radiation treatment is an important technique for treating masses in the sellar and parasellar regions such as meningiomas, adenomas, craniopharyngiomas, and others. Such local radiation treatment may negatively affect mnestic function, which, given the benign nature of the lesions, may be of long-term importance. Additional studies to determine tolerated doses to the hippocampus are warranted. Taking into account the data already available, the hippocampus should be considered as a critical structure also in the local irradiation of intracranial neoplasms.
